# Multi-Colored Light-Emitting Electrochemical Cells Based on Thermal Activated Delayed Fluorescence Host

**DOI:** 10.1038/s41598-017-01812-2

**Published:** 2017-05-08

**Authors:** Jiang Liu, Jorge Oliva, Kwing Tong, Fangchao Zhao, Dustin Chen, Qibing Pei

**Affiliations:** Department of Materials Science and Engineering, University of California, Los Angeles, California 90095 USA

## Abstract

Light-emitting electrochemical cells (LECs) with the thermally activated delayed fluorescence(TADF) host and phosphorescent guests were fabricated using solution process. It is demonstrated for the first time that TADF, a well-known phenomenon that helps to increase electroluminescence efficiency by harvesting excitons from triplet states, is used as a host in LECs. Devices with green, yellow, red and warm white emissions were fabricated, with the best devices showing more than 7000 cd/m^2^ stable emission and a peak efficiency over 7 cd/A. Under high voltage stress, a burst of extremely high luminance of over 30,000 cd/m^2^ was observed. All these LEC devices are extremely simple with only one active layer. Thus, our results could pave way to produce low- cost light source with high luminance, using TADF molecules.

## Introduction

During the electroluminescence process in an organic light-emitting diode (OLED), electrons and holes recombine and generate singlet and triplet excitons in a 1:3 ratio according to spin statistics^1^. Only the 25% of singlet can be converted to light in a fluorescent OLED^2^. To harvest the 75% triplet excitons, several methods have been developed^3^, among which the utilizations of novel thermally activated delayed fluorescence (TADF) materials have been intensively investigated in recent years^4^.

**Figure 1:**
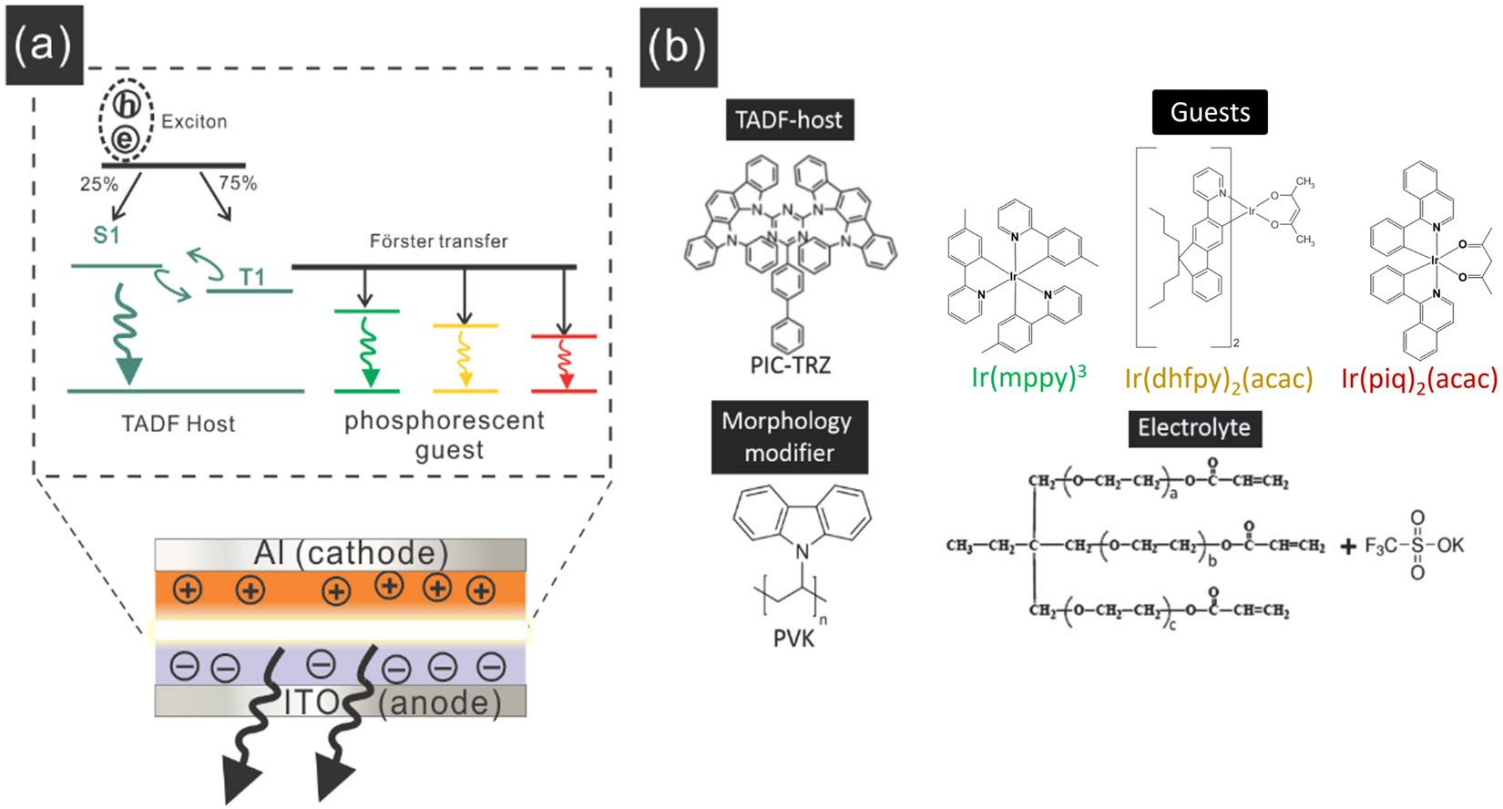
(**a**) Schematics for the emission mechanisms in TADF-LECs, (**b**) chemical structure of the TADF host, P.D. molecules, morphology modifier and electrolyte employed in this work.

Metal-free TADF molecules offer unique optical and electronic properties arising from their molecular design that includes both electron donor and acceptor units in a single molecule. The resulted very small energy split (ΔE_st_1) between the singlet excited state(S_1_) and excited triplet states(T_1_) in TADF molecules, leads to efficient inter-transition from T_1_ to S_1_; see energy transfer mechanism in Fig. 1a. Therefore, they have been widely used as dopants in highly efficient OLEDs. Another appealing property of TADF molecules is their intrinsic bipolar charge transport ability, i.e. they conduct both electrons and holes^5, 6^, which makes them ideal host materials. Several groups have employed TADF host in combination with phosphorescent dopants (P.D.) in OLEDs and demonstrate comparable performance to conventional OLEDs with fluorescent host doped with P.D.^4, 7, 8^. Compared to fluorescent host, TADF host also enjoy the following advantages: they show a smaller turn-on voltage due to their small bandgap, and require smaller amount of P.D.^9, 10^.

Alternative devices to OLED device have been developed such as quantum dot perovskite LED^11, 12^ and solid state light-emitting electrochemical cell (LEC)^13–16^. LECs have emerged as a promising candidate for next-generation lighting applications, such as light-emitting fibers^8^ and emissive papers^17^, due to its simplicity in device structure and their potential to realize large scale printed lighting source^18–21^. An LEC, in its simplest form, contains a light emitting active layer sandwiched between two charge injection electrodes (see Fig. 1a). The active layer is usually a mixture of light emitting molecular and an electrolyte. An LEC can be seen as a dynamic organic p-i-n junction that is self-assembled through electrochemical doping during operation^22, 23^. LECs possess the processing advantage over OLED, but the efficiency, as a trade-off, has never reached the level of OLED devices. The most efficient materials combination in OLED–fluorescent hosts with P.D.—has failed to reproduce high performance in LECs. There are two drawbacks for using the OLEDs’ fluorescent hosts in LECs: first, they have a large bandgap which is usually larger than the electrochemical stability window; secondly, they are usually p-type semiconductors and shows a weak n-doping. Therefore, the reported LEC with fluorescent hosts: P.D. combination are instable and showed a low efficiency^24–26^. On the other hand, TADF compounds might serve as a more suitable host for LEC, because they have a smaller bandgap, and could conduct both electron and holes and thus is capable of being n-doped and p-doped^26^. The motivation behind our research is to explore the TADF materials as a host in an LEC configuration. We will show that in a TADF-LEC which consists of a TADF host, different P.D.s and an electrolyte, is fully functional with decent efficiency. This device is able to emit red, green, yellow, red with stable luminance over 6000 cd/m^2^ and peak efficiency above 7 cd/A. We also demonstrate a white-light LEC made from blue-green emission TADF host combined with green and red dopants.

## Results

The TADF host is 2-biphenyl-4,6-bis(12-phenylindolo[2,3-a]carbazole-11-yl)-1,3,5-triazine (PIC-TRZ). PIC-TRZ is the first TADF material that has been synthesized by Adachi and co-workers, it shows a small singlet-triplet energy difference (E_st_) of 0.15 eV and a high triplet to singlet conversion rate of 29%^27^. The green, yellow and red dopants are tris[2-(p-tolyl)pyridine]iridiuM(III) (Ir(mppy)_3_ or G.D.), bis(m2-(9,9-dihexylfluorenyl)-1-pyridine)(acetylacetonate)iridium (III) (Ir(dhfpy)_2_(acac) or Y.D.), and Bis(1-phenylisoquinoline)-(acetylacetonate) iridium (III) (Ir(piq)_2_(acac) or R.D.), respectively. The electrolyte is a mixture of an ion conductor ethoxylated trimethylolpropanetriacrylate (ETPTA), and a salt potassium trifluoromethanesulfonate(KCF_3_SO_3_). ETPTA is chosen as the ion conductor because of its polymerizable properties, which is crucial to form a stable LEC to enhance the lifetime of the devices^28^. The structures for the TADF host, fluorescent dopant and electrolyte are depicted in Fig. 1b.

### Photoluminescence Characterization

**Figure 2:**
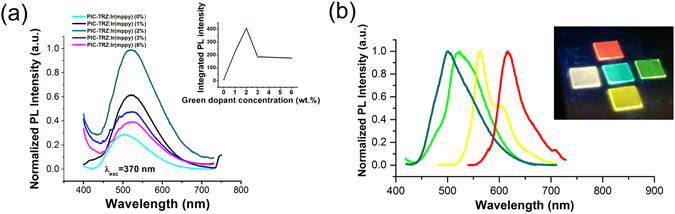
PL characterizations. (**a**) PL response with PIC:TRZ doped with different concentration of G.D.(x%), x = 0, 1, 2, 3 and 6. Inset shows the integrated PL intensity vs. concentration%. (**b**) PL spectrum of PIC:TRZ doped with different dopants.

First the energy transfer from the TADF host to P.D. by photoluminescence (PL) experiments were examined to determine an optimum concentration between TADF host and P.D. Thin films composed of PIC:TRZ: G.D.: ETPTA: KCF_3_SO_3_ in a weight ratio of 100:x, with x = 0, 1, 2, 3, and 6 were illuminated by 370 nm UV light and the PL response was recorded and shown in Fig. 2a. PL measurement at 370 nm wavelength was chosen because both PIC:TRZ and G.D. showed active response to this wavelength, as seen in excitation spectrum of Figure S1 (see supplementary information). As can be seen in Fig. 2a, the PL response of the host-only device (x = 0) shows a typical PIC-TRZ spectrum with a peak at 510 nm, while the PL spectrum for host: guest mixture (x = 1, 2, 3, 6) all showed guest-only spectrum with a peak at 525 nm, indicating a complete energy transfer from the host to guest. Förster resonance energy transfer occurs here because the PL energy of PIC-TRZ can be absorbed by each dopant as observed in Figure S2. It also showed in the inset of Fig. 2a that, PL intensity peaks at 2% of guest concentration. The intensity drops for concentrations above 2% probably due to the quenching concentration effect^29^. We did the same experiment for Y.D. and R.D., and they also showed an optimum concentration between 1.5% to 2.5%. Therefore, TADF-LECs with 2% of dopant concentration was chosen in the following electrical experiment. The PL spectra of PIC:TRZ: dopants (2%): ETPTA: KCF_3_SO_3_ with different dopants in solid films are shown in Fig. 2b, with the photos of these samples under UV light at 370 nm are shown in the inset. Figure 2 also confirms that 2% of doping concentration is sufficient to quench all the host emission, indicating a complete energy transfer from TADF host to P.D.s at 2% of doping concentration.

### TADF-LEC Fabrication

After confirming from the PL measurement that PIC-TRZ is transferring energy fully to P.D. in an ionic environment, the PIC-TRZ: P.D.: electrolyte mixture was investigated in LEC configuration for electroluminescent measurements (EL). Direct coating of this mixture onto a glass substrate would contain pinholes due to the small molecular weight(MW) of the materials. Therefore, a small amount of poly(9-vinylcarbazole) (PVK) with 25,000 MW was added into the mixture to increase the mixture’s film forming ability (see molecule structure in Fig. 1b). The effect of PVK will be discussed in details in a subsequent section. After optimization, the final weight ratio between PIC:TRZ: P.D.: ETPTA: KCF_3_SO_3_: PVK were 100:2:20:2:5. This mixture was dissolved in a common solvent cyclopentanone, with the a PIC:TRZ concentration of 65 mg/mL. The solution is then spincoated with 3000–3500 rpm onto ITO-glass substrate, forming a 120 nm solid film. Subsequently, a layer of 80 nm aluminum (Al) was thermal-evaporated on top of the active materials to finalize the TADF-LEC at a base pressure of 2 × 10^−6^ Torr.

### Electroluminescent Characterizations of the TADF-LECs

**Figure 3:**
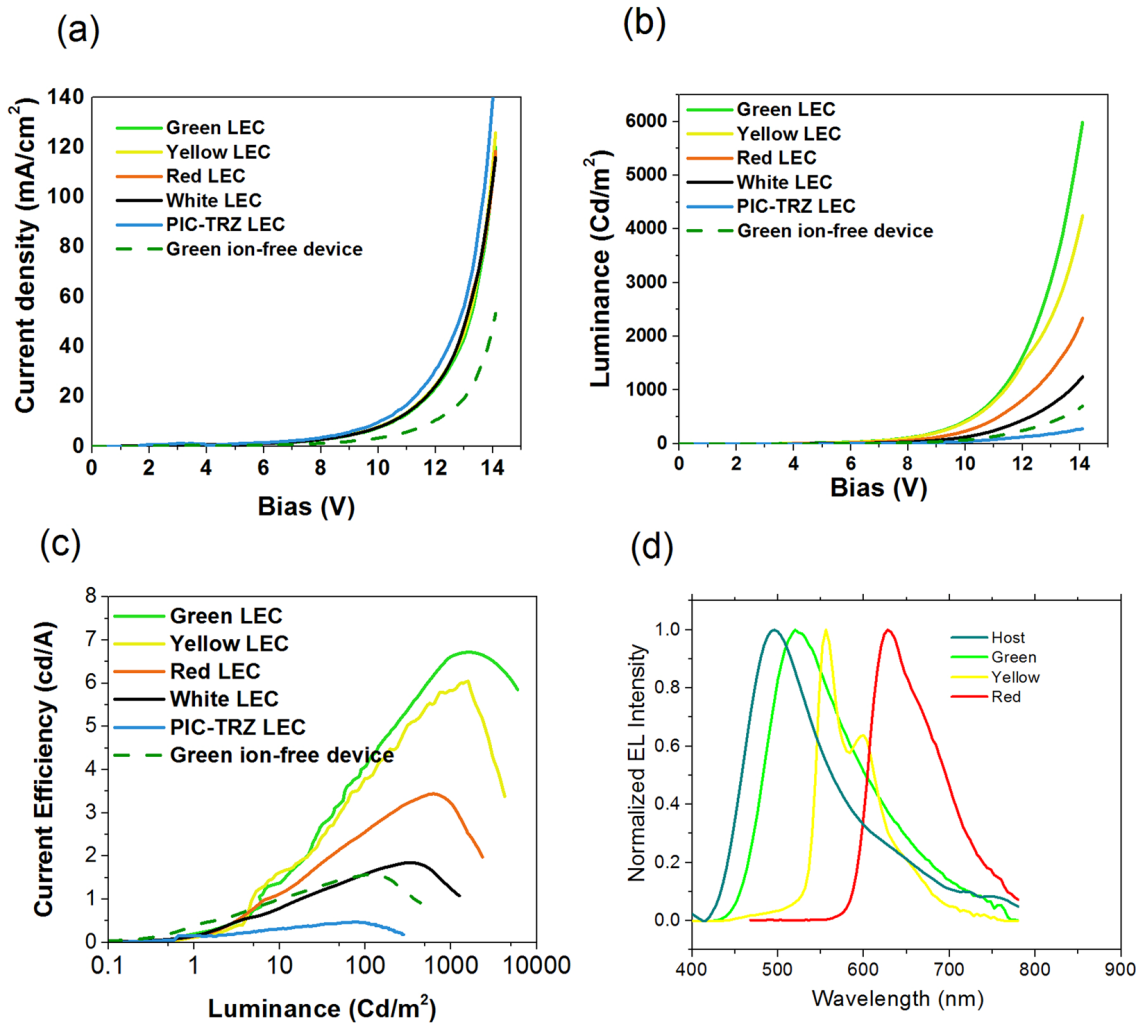
EL characterizations. (**a**) Current density and (**b**) luminance at different applied potential; (**b**) current efficiency at different luminance; (**d**) EL spectra for different devices.

The as-fabricated TADF-LEC devices with different dopants (G.D., Y.D. and R.D.) were then characterized electrically and optically, as shown in Fig. 3. In general, the device was first held at 8 V between ITO and Al electrodes for about 30 s to achieve a relatively stable electrical current and luminance. Within this period, the ions would electrochemically react with PIC-TRZ, forming a p-doped region near ITO (anode) and an n-doped region near Al (cathode). This anode/n-doped region/PIC-TRZ: P.D/p-doped region/cathode resembles the structure of a p-i-n OLED. Indeed, after biasing, the current density (J-V, Fig. 3a) and luminance (L-V, Fig. 3b) verse V showed typical diode rectification characteristics with the voltage (V) sweeping from 0 V to 15 V. The turn-on voltage for all devices was around 3 V, which is similar to that of OLEDs made from PIC-TRZ^10^. Note that the LEC without dopant shows a maximum current efficacy (CE_max_) of 0.65 cd/A, and maximum luminance (L_max_) of 700 cd/m^2^. While the TADF-LECs with green, yellow and red dopants show a maximum CE of 6.8, 5.6, 3.2 cd/A, and a maximum L of 6200, 4800 and 3100 cd/A, respectively (summarized in Table 1). This indicated that the P.D. has greatly improved light-emitting ability and efficiency of the device by harvesting the triplet excitons. Further, the introduction of dopants decreased J, suggesting that the presence of dopants changes the charge transport properties of the active layer. As can be seen from Fig. 3c, CE rolls off at high L, also similar to conventional OLEDs. The EL spectra of these devices are shown in Fig. 3d, showing identical emission spectrum as shown in OLED devices.

**Table 1:** Electrical properties from different TADF-LECs.

Dopant	λ_Peak_ (nm)	Max L (cd m^−2^)	MAX C.E (cd A^−1^)	C.E at 1000 cd m^−2^ (cd A^−1^)
None	495	294	0.5	N/A
Green (2%)	541	6000	7.1	6.3
Yellow (2%)	559	4243	6.1	5.5
Red (2%)	630	2360	3.5	2.8
White:	513; 630	1220	1.8	1.0

A green device containing no ions was also fabricated and measured under the identical conditions to the green TADF-LEC, with the electrical and optical characterization shown as the dash line in Fig. 3a–c. The ion-free device having a structure of ITO/PIC:TRZ: G.D.: ETPTA: PVK /Al can be seen as an OLED with no electron/hole injection and transport layer, and it exhibited a much lower J and L, comparing to the LEC device containing ions. Moreover, the ion-free device showed a maximum current efficiency of 1.4 cd/A, which is about 80% lower than that of the ion-containing devices. This can be explained by the following: in the ion-containing device, the addition of the ionic moieties induces the p-doping of organic semiconductor near anode and n-doping near cathode, *in-situ* enabling an p-i-n junction formation^28, 30, 31^. The p- and n- doped organic semiconductor becomes more conductive than the intrinsic one^32^, resulting a more conductive device and thus a higher current than the ion-free device; moreover, the ion-induced doping provides ohmic contact between the semiconductor and charge-injection electrodes and thus lowers the driving V and increases the efficiency.

### White TADF-LEC

**Figure 4:**
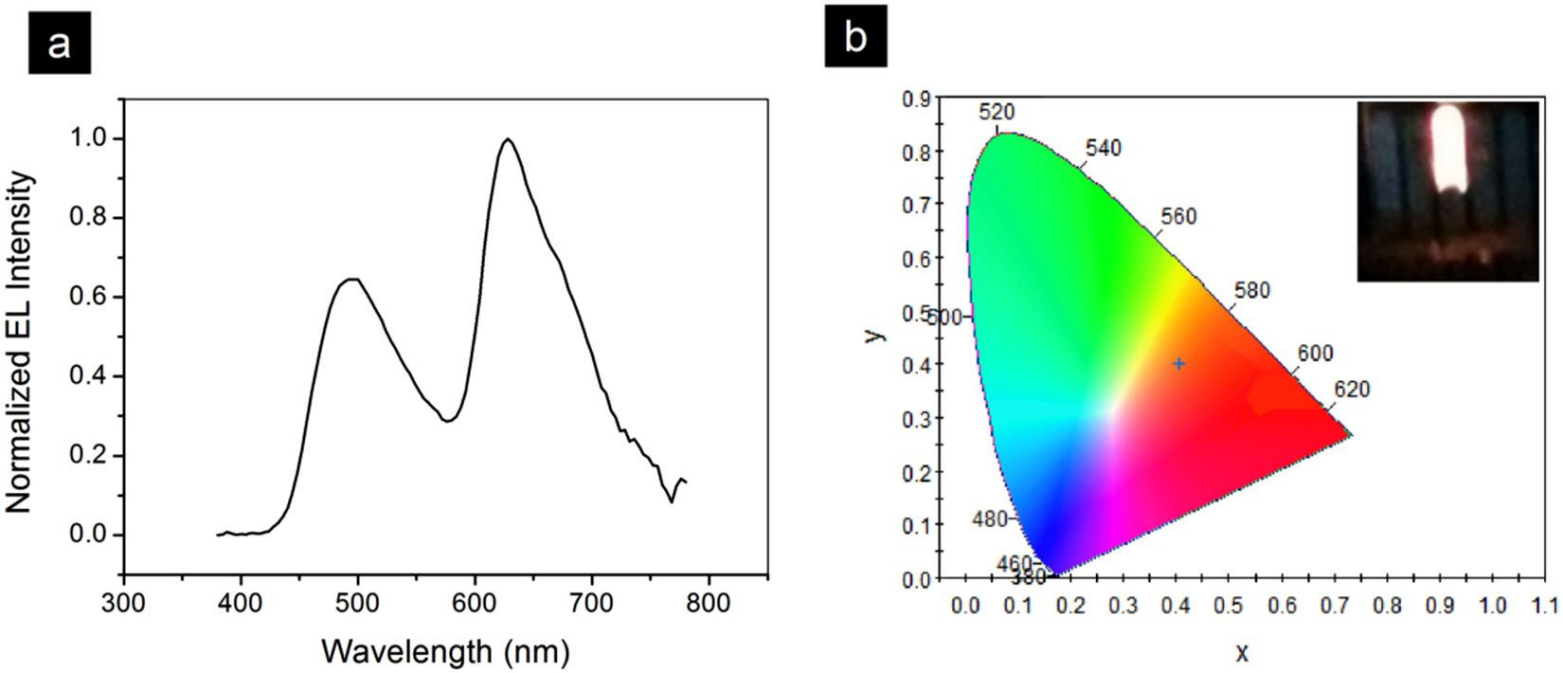
(**a**) EL spectrum for white LEC; (**b**) CIR coordinates for the white light emission. The inset shows a photo of the device emitting white light.

White light emitting devices made from organic materials are of great interest^33–38^, because of its potential in low-cost, large-area lighting applications. We combined PIC:TRZ TADF host which emits blue-green light, to very small amount of green and red emitting dopant for white-light LEC (TADF-WLEC). With a relatively small amount of dopants in the mixture, only part of the energy received from the host is transferred to the guest. As a result, part of the TADF-host would still emit blue-green light, while G.D and R.D emits green and red light, respectively. Therefore, a properly mixed emission profile from these molecules would generate white light. After trying different concentration of dopants, we have obtained an optimized white emitting device with the composition of PIC:TRZ: G.D.: R.D.: ETPTA: KCF_3_SO_3_: PVK in a weight ratio of 100:0.04:0.02:20:2:5. The J-V and L-V characteristics are shown in Fig. 2b and c, and the light emission spectrum is shown in Fig. 4a. As can be seen in Fig. 4b, the white color has a CIE color index of (0.39, 0.37), a warm color temperature of 3567 K, and a color rending index (CRI) of 61% due to a lack of deep blue emission. CE_max_ and L_max_ is 1.8 cd/A, and 1220 cd/m^2^, respectively.

## Discussion

### The effect of PVK

**Figure 5:**
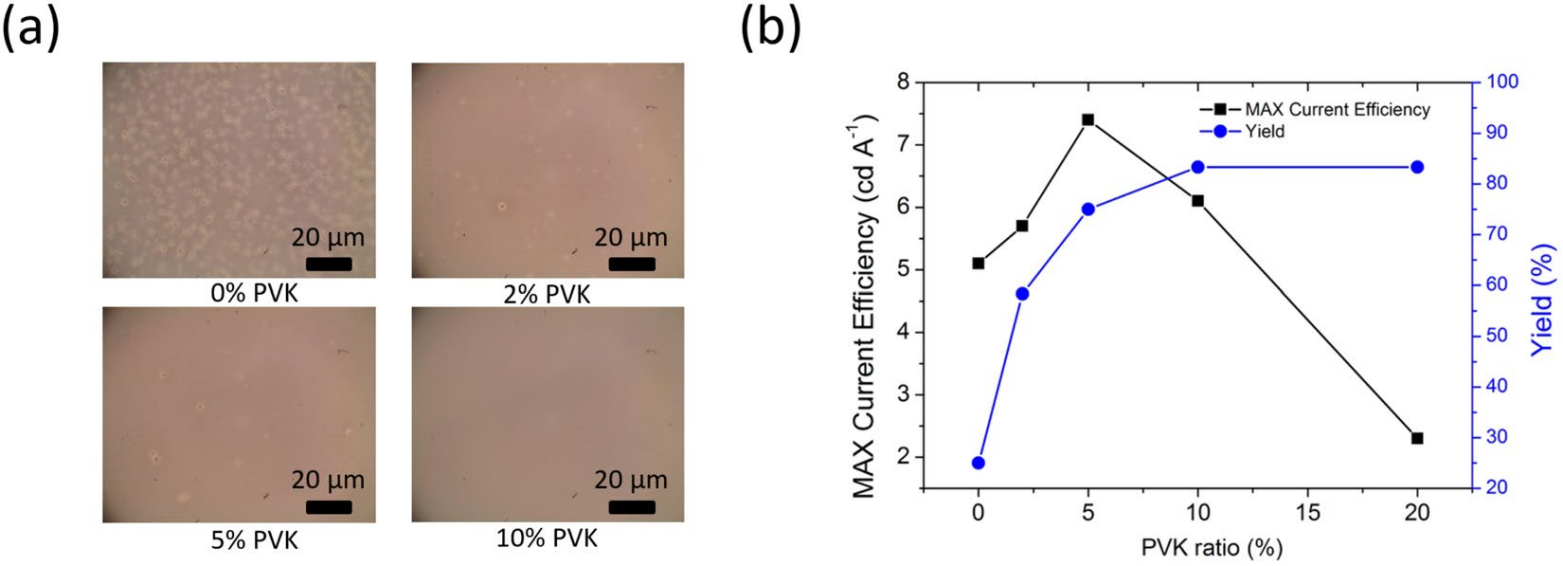
(**a**) Optical microscopic image of as- casted film consisted of PIC-TRZ: G.D.: electrolyte: PVK (0–10%); (**b**) Dependence of C.E_max_ and yield vs. PVK ratio.

As mentioned previously, the active components are small molecules and electrolytes. Both of these components have small MWs, and a polymer (PVK) with a relatively large MW was added into the mixture to improve film-forming properties. The film casted by PIC-TRZ: G.D.: electrolyte with the addition of PVK (0, 2, 5 and 10% in weight) were imaged by optical microscope and displayed in Fig. 5a. The film with 0% PVK showed a large number of pinholes with diameters around 5–10 µm; as the weight percentage of PVK was increased to 2%, the number of pinholes dramatically decreased; 5% of PVK would further reduce the pinhole population; and 10% addition of PVK would almost eliminate the pinholes. (AFM image, however, doesn’t reveal much difference in films due to the large size of pinholes; see Figure S3 in supplementary information) This difference in film-forming abilities with different proportion of PVK greatly impact the device characteristics, in terms of peak efficiency and production yield. Green TADF-LEC devices made with different weight percentages of PVK were measured with their performance plotted in Fig. 5b. The parameters were averaged with a group of 12 devices. As can be seen in the figure, the device without PVK had a very low yield of around 25% and a low C.E. of 2 cd/A. The addition of a moderate amount of 2–5% PVK effectively increased up to 75% yield and 7.1 cd/A C.E_max_. Adding larger amount (10–20%) of PVK further improves the yield but deteriorate the device efficiency. The decrease in device performance with high amount of PVK was probably due to the relatively low and unbalanced charge transport properties. Addition of 5% PVK has been proven to be the optimum ratio with peak device performance and acceptable production yield.

### Stability study

**Figure 6:**
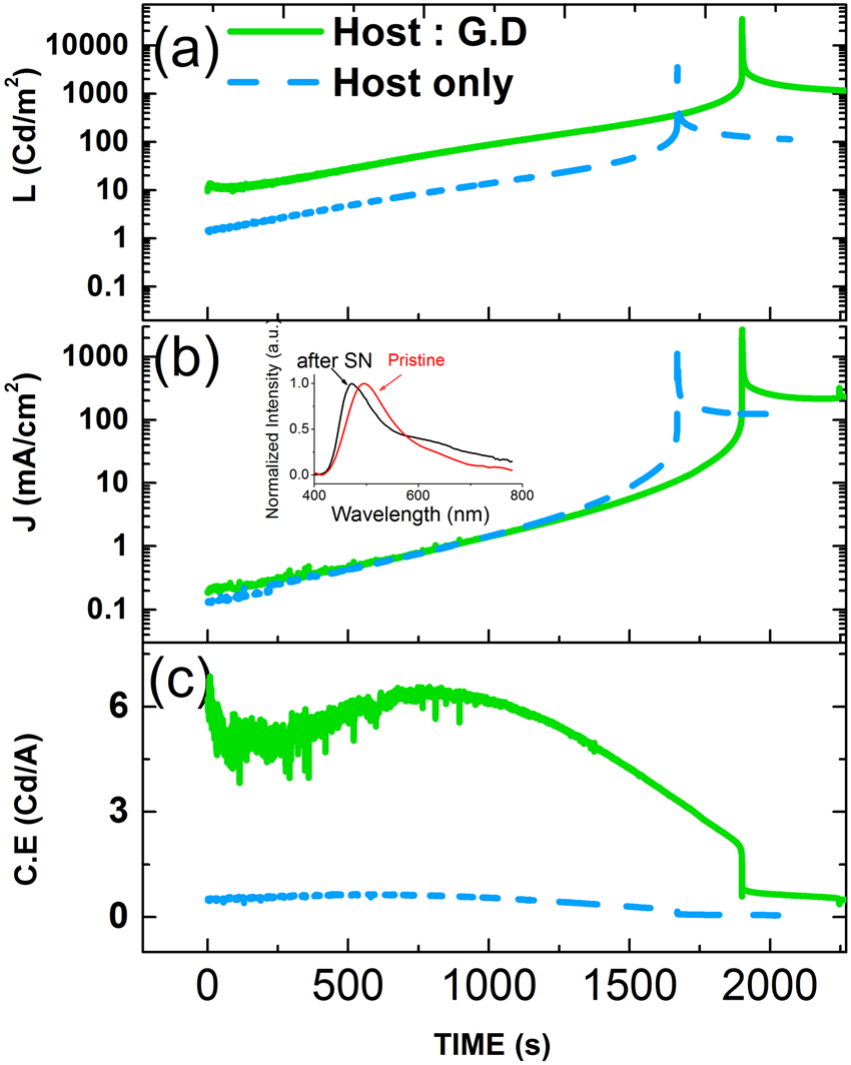
The change of (**a**) luminance, (**b**) current density and (**c**) current efficiency during the burst of luminance. Inset shows the spectrum change before and after the emission burst.

To further study the stability of the TADF-LEC devices, the host-only TADF-LEC device was stressed at a relatively high voltage (14 V) for more than 2000 s (Fig. 6a). During the first 1700 s, J and L gradually increased, ascribed to the drop of device resistance(R_s_) during continuous electrochemical doping. After 1800 s, a more than 10-fold dramatic raise of both J and L was observed, followed by a rapid decrease of J and L. This momentary burst lasted about 1 s. After that, the device continued to emit light at very low C.E. A change of spectrum from 495 nm to 447 nm was also seen. This blue shift of 48 nm in the emission spectrum indicates the widening of bandgap, as a result of change in molecular structures. The LEC device was then left open-circuit for 24 hours, and emission was measured to still peak at 447 nm, indicating that this change in chemical structure is irreversible.

A similar burst phenomenon was also observed in host: P.D. device. It is worth noting that, the peak luminance for green LEC reached 35,000 cd/m_2_. This is the brightest luminance from LECs ever reported, to the best of authors’ knowledge.

This momentary brightness burst is not observed in other polymer-based LECs or ITMC-based devices in our laboratory, nor has been reported in literatures^39, 40^. It was also observed in LECs using another TADF molecule as host, 2,4,5,6-tetrakis(carbazol-9-yl)-1,3-dicyanobenzene;(4r,6r)-2,4,5,6-tetra(9H-carbazol-9-yl)isophthalonitrile (4CZIPN). This process in TADF-LECs is speculated to be attributed by the thinning of the p-i-n junction, with a centered light emission region as a result of balanced electron and hole transport in TADF compounds. In addition, freezing the LEC during the burst process (before it decades) would be of great interest for providing a low-cost and bright light source, given its very simple structure.

## Conclusions

To conclude, we have demonstrated an ultra-simple, single-layer, light-emitting electrochemical cell based on thermal activated delayed fluorescence host. The active layer is a solution processed mixture of a host materials possessing TADF property, various different dopants, an electrolyte, and a morphology improver PVK. By mixing the different dopants at different ratios, different color emissions were achieved, including green, yellow, red and a warm white light emission. The host-only blue-green device emits lower than 300 cd/cm^2^ with a maximum efficiency of 0.5 cd/A. While the green, yellow, red, and white LECs emits maximum 6000, 4200, 2300, 1200 cd/m^2^ with a peak efficiency of 7.1 cd/A, respectively. The green device also emits 35,000 cd/m^2^ for a momentary period, which is the brightest LEC to the best of the authors’ knowledge. Taking advantages of TADF molecules’ optical and transport properties, TADF-LECs outperform LECs with phosphorescent hosts, in terms of efficiency and maximum brightness^26, 41^. Moreover, the TADF-LEC possesses a simple single-layer structure of solution-processed materials, which could be potentially used to produce cost-efficient, fully printed lighting source with high luminance.

## Experimental

### Materials

The TADF host 2-biphenyl-4,6-bis(12-phenylindolo[2,3-a]carbazol-11-yl)-1,3,5-triazine (PIC-TRZ) was purchased from Lumtec, while the guests: tris[4-(*o*-tolyl)pyridine]iridium(III) [Ir(mppy)3] (green dopant), Bis(2-(9,9-dihexylfluorenyl)-1-pyridine)(acetylacetonate)iridium (III) (yellow dopant) and Bis(1-phenylisoquinoline)-(acetylacetonate) iridium (III) (red dopant) were acquired from American Dye Source. The ionic source Potassium trifluorinate (KCF_3_SO_3_) and Polyvinylcarbazole were obtained from Sigma-Aldrich. Sr9035 (ionic conductor) was provided by Sartomer.

### Solution preparation

PIC:TRZ: P.D.: ETPTA: KCF_3_SO_3_: PVK were mixed in cyclopentanone with a weight ratio of 100:2:20:2:5. The concentration of PIC-TRZ is 65 mg/mL.

#### PL samples

The as prepared solution was spun onto glass substrate at 3000 rpm for 30 s.

#### TADF-LEC Devices

The TADF-LEC Devices were fabricated in a vertical structure, with indium tin oxide (ITO) as the anode and aluminum as cathode, the single layer of active light-emitting materials is sandwiched in between these two electrodes, as seen in the bottom of Fig. 1a. The active layer is a solid mixture which consists of the following components: a TADF host 2-biphenyl-4,6-bis(12-phenylindolo[2,3-a]carbazole-11-yl)-1,3,5-triazine (PIC-TRZ), various P.D., an ion conductor, and poly(9-vinylcarbazole) (PVK) as a surface modifier. The green, yellow and red dopants were tris[2-(p-tolyl)pyridine]iridiuM(III) (Ir(mppy)_3_), bis(m2-(9,9-dihexylfluorenyl)-1-pyridine)(acetylacetonate)iridium (III) (Ir(dhfpy)_2_(acac)), and Bis(1-phenylisoquinoline)-(acetylacetonate) iridium (III) (Ir(piq)_2_(acac)), respectively. The chemical structures of all those compounds are shown in Fig. 1b. A typical device was fabricated as follows: PIC:TRZ: P.D.: ETPTA: KCF_3_SO_3_: PVK were mixed in cyclopentanone with a weight ratio of 100:2:20:2:5 are dissolved in a common solvent cyclohexanone, with a concentration of PIC-TRZ being 65 mg/mL.

### Optical Characterization

Photoluminescence (PL) characterization was performed using a Xenon lamp of 75 W excitation power. The fluorescence emission was analyzed using a Photon Technology International fluorometer. The system was PC controlled and the emission obtained with PTI software. All optical characterization was performed at room temperature and thin films of each material were made by spin coating a mixture of PIC:TRZ: P.D. (2% wt) in cyclohexanone. Special care was taken to keep the alignment of the setup in order to compare the emission intensity among different samples. The Absorption spectra of the mixtures were recorded in the range of 200 to 700 nm using a Shimadzu UV-1700 spectrophotometer.

### Morphology Characterization

Optical microscopy was performed on a TFProbe micrscope under darkfield imaging at 80x magnification using a polarizer setup. Surface topography was carried out on a Dimension Fastscan Scanning Probe Microscope (SPM) from Bruker. All images were taken under ambient conditions.

### Electrical Characterization

The current-voltage, Luminance- voltage and efficiency-voltage curves were obtained using a Labview software which synchronizes a Keithley 2400 source meter and a calibrated silicon photodetector. The Electroluminescence spectra, color temperature and CIE coordinates were measured utilizing a photoresearch PR-655 spectroradiometer. All measurements were made under N_2_ atmosphere.

## Electronic supplementary material

Supplementary Information
