# Supplementary material for: Multi-Colored Light-Emitting Electrochemical Cells Based on Thermal Activated Delayed Fluorescence Host
Source: Sci Rep. 2017 May 8;7:1524. doi: 10.1038/s41598-017-01812-2 (PMC5431554; doi:10.1038/s41598-017-01812-2)
Supplement: Supplementary file 1 — Supplementary Information [file 41598_2017_1812_MOESM1_ESM.pdf]

Supplementary Information for

" Multi-colored light-emitting electrochemical cells based on thermal activated delayed fluorescence host"

Jiang Liu, Jorge Oliva, Kwing Tong, Fangchao Zhao, Dustin Chen and Qibing Pei

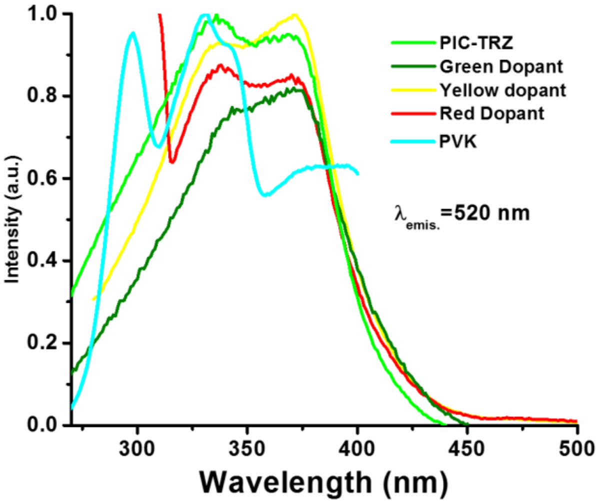

Figure S1. Excitation spectrum of dopants employed in this work.

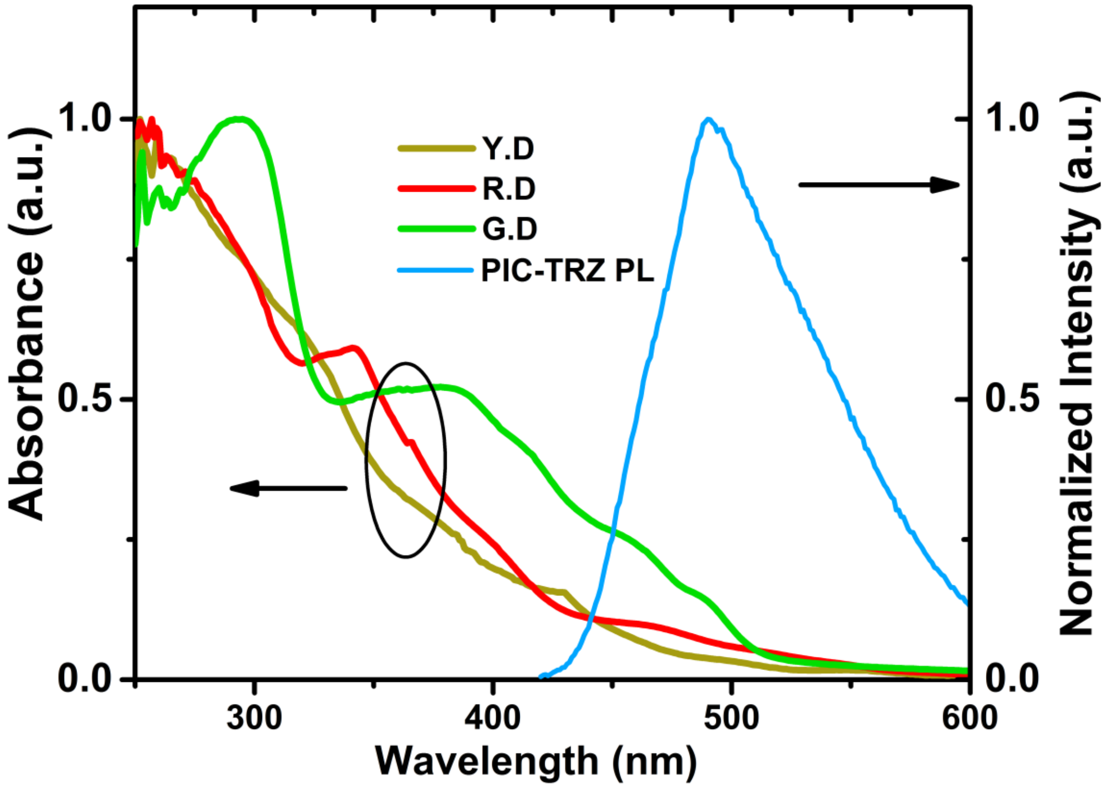

Figure S2. The absorbance of dopants and the photoluminesecence (PL) intensity of the host.

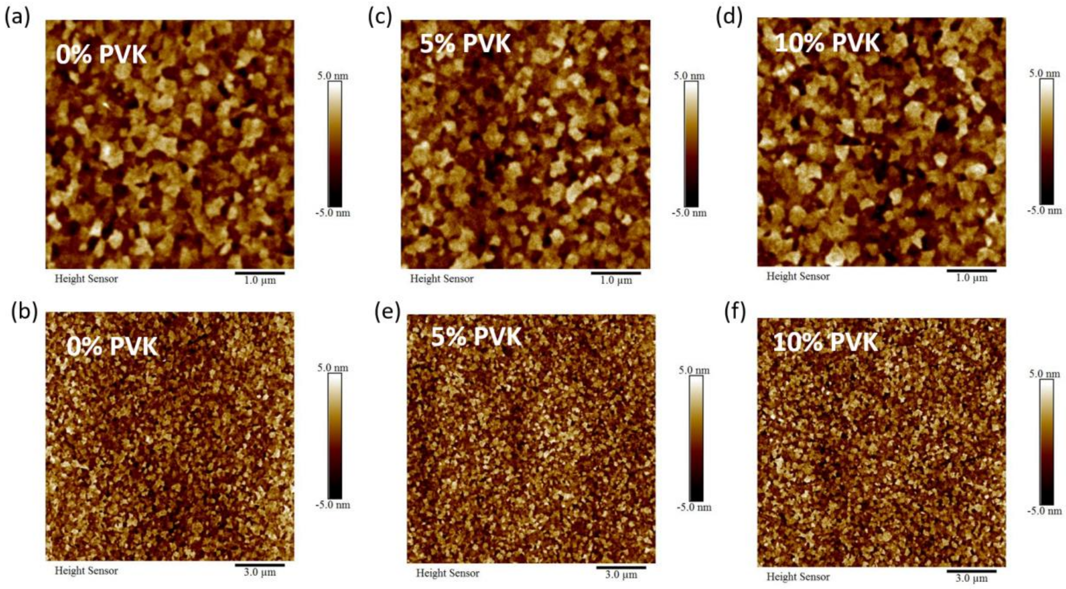

Figure S3. Atomic force microscopic image in different scales for samples with different amount of PVK addition.
